# Hypermethylation of the 16q23.1 Tumor Suppressor Gene *ADAMTS18* in Clear Cell Renal Cell Carcinoma

**DOI:** 10.3390/ijms16011051

**Published:** 2015-01-05

**Authors:** Ben Xu, Lian Zhang, Cheng Luo, Yan Qi, Yun Cui, Jian-Ming Ying, Qian Zhang, Jie Jin

**Affiliations:** 1 Department of Urology, Peking University First Hospital and Institute of Urology, Peking University, National Urological Cancer Center, 8 Xishiku Street, Xicheng District, Beijing 100034, China; E-Mails: xuben_pku@sina.com (B.X.); hays0324@163.com (L.Z.); luocheng-927@163.com (C.L.); cuiyun_pku@sina.com (Y.C.); 2 Department of Urology, Affiliated Hospital of Taishan Medical University, Tai’an 271000, China; E-Mail: sunyingqiyan@126.com; 3 Department of Pathology, Cancer Hospital & Cancer Institute, PUMC, Chinese Academy of Medical Sciences, Beijing 100021, China; E-Mail: jmying@hotmail.com

**Keywords:** hypermethylation, *ADAMTS18* gene, clear cell renal cell carcinoma, tumor suppressor gene

## Abstract

To identify tumor suppressor genes (TSGs) silenced by hypermethylation and discover new epigenetic biomarkers for early cancer detection. *ADAMTS18*, located at 16q23.1, has been reported to be a critical TSG in multiple primary tumors; however, this has not yet been verified in clear cell renal cell carcinoma (ccRCC). We explored epigenetic alterations in this gene in ccRCC and analyzed possible clinicopathological associations. We examined *ADAMTS18* gene expression and methylation by semi-quantitative reverse transcription PCR (RT-PCR) and methylation-specific polymerase chain reaction (MSP) in 5 ccRCC-derived cell lines before and after treatment with 5-aza-2'-deoxycytidine (5-AzaC). MSP was further performed for 101 ccRCC primary tumors and 20 adjacent normal tissues. Some cell lines and specimens were examined by subsequent bisulfite genomic sequencing (BGS) and real-time PCR. Further, we analyzed the relationship between the *ADAMTS18* gene methylation and clinicopathological features, including short-term disease-free survival (DFS), in patients with ccRCC. *ADAMTS18* down-regulation and hypermethylation were detected in the ccRCC-derived cell lines using RT-PCR and MSP. Treatment with 5-AzaC reversed the hypermethylation of the *ADAMTS18* gene and restored its expression. Hypermethylation was further detected in 44 of 101 (43.6%) primary tumors and 3 of 20 (15.0%) adjacent normal tissues. However, a significant difference between both groups was observed (*p* = 0.02). BGS analysis and real-time PCR were subsequently performed to confirm the results of RT-PCR and MSP. Furthermore, the methylation status of *ADAMTS18* was not significantly associated with gender, age, location, tumor diameter, pathological stage, nuclear grade or short-term DFS in patients with ccRCC (*p* > 0.05). The *ADAMTS18* gene is often down-regulated by hypermethylation in ccRCC-derived cell lines and primary tumors, indicating its critical role as a TSG in ccRCC. We conclude that *ADAMTS18* gene hypermethylation may be involved in the tumorigenesis of ccRCC and may serve as a novel biomarker for this disease.

## 1. Introduction

Clear cell renal cell carcinoma (ccRCC) is the most common type of RCC, and it often exhibits an aggressive phenotype, including frequent metastasis to distant organs and resistance to therapeutic approaches including chemotherapy and radiotherapy [1]. An increasing number of studies have demonstrated that the inactivation of tumor suppressor genes (TSGs) is a frequent event involved in the tumorigenesis of ccRCC as a result of epigenetic abnormalities in DNA methylation [2].

Previous research has demonstrated that hypermethylation of the core promoter region within CpG islands is associated with the loss of transcription of classical TSGs in multiple tumor types [3]. Currently, a large number of TSGs in a wide range of cancers have been found to be inactivated by hypermethylation of the promoter [4,5], and much of this hypermethylation occurs in the context of 5'-CpG islands, which are regions of dense accumulation of CG dinucleotides found in approximately 70% of mammalian protein-coding genes [6]. Therefore, it has been proposed that the hypermethylation status of specific TSGs is a potentially sensitive marker that may be used in ccRCC diagnosis and prognosis prediction [2,7]. As a matter of fact, several previously identified classical TSGs, such as *VHL*, *CDKN2A/p16INK4a*, *CDH1/E-cadherin* and *SDHB*, are known to be hypermethylated in subsets of sporadic ccRCC [8]. Given that in a recent literature review only 43 genes have been reported that are methylated in >20% of RCC, it significantly expands the catalog of methylated RCC genes that can be investigated for application as potential biomarkers for the detection, diagnosis, prognostication and therapy of RCC [9]. We have previously identified the “frequently” methylated TSGs *DLEC1* (31%) and *DLC1* (35%) in RCC primary tumors [10,11]. And at least 8 gene promoters have been reported recently to be frequently methylated in RCC: *ATP5G2* (36%), *CORO6* (22%), *KLHL35* (39%), *QPCT* (19%), *SCUBE3* (19%), *ZSCAN18* (32%), *CCDC8* (35%) and *FBN2* (34%) [12]. However, the methylation level of these genes is still low; thus, additional TSGs with higher methylation levels in ccRCC still need to be confirmed.

The *ADAMTS18* gene, which is located in the 16q23.1 region and encodes a disintegrin and metalloproteinase with thrombospondin motifs, belongs to the *ADAMTS* family, which is often involved in ectodomain shedding or the activation of diverse cell surface molecules, including growth factors and adhesion receptors [13]. Jin, H* et al.* [14], validated the finding that the *ADAMTS18* gene is frequently hypermethylated in a variety of tumor tissues, including oesophageal squamous cell carcinoma (24/46, 52%), nasopharyngeal carcinoma (30/43, 70%), hepatocellular carcinoma (6/20, 30%), breast carcinoma (5/21, 24%), cervical carcinoma (5/8, 63%) and other carcinomas. Consequently, this hypermethylation may be a driving mechanism for *ADAMTS18* gene silencing in a wide range of tumors. No studies have yet determined whether there is an association between the methylation status of *ADAMTS18* and urological tumors, including ccRCC, bladder cancer and prostate cancer.

The current study represents the first exploration of *ADAMTS18* gene hypermethylation in ccRCC-derived cell lines and primary tumors. The relationship between *ADAMTS18* gene methylation status and clinicopathological features in patients with ccRCC was also analyzed, which has not been previously studied or reported to our knowledge. This investigation provides valuable information that can be used to determine the utility of the *ADAMTS18* gene as a potential novel biomarker for ccRCC diagnosis and prognosis and a potential ccRCC therapeutic target.

## 2. Results

### 2.1. Down-Regulation of ADAMTS18 Gene Expression in Clear Cell Renal Cell Carcinoma (ccRCC)-Derived Cell Lines

**Figure 1:**
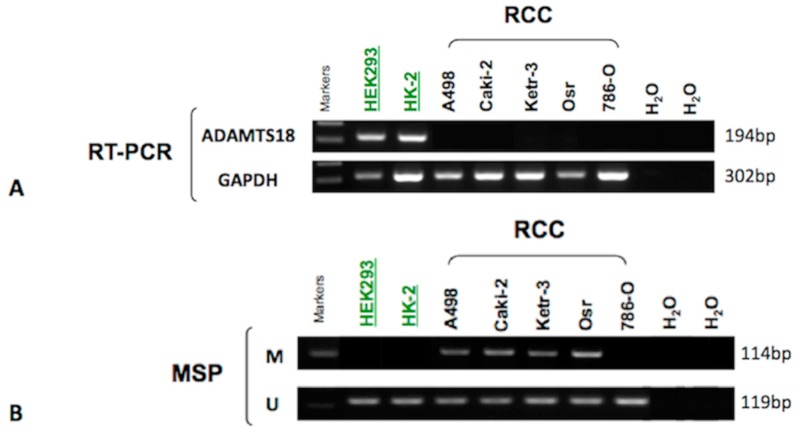
Analysis of the *ADAMTS18* gene in various cell lines. (**A**) *ADAMTS18* gene expression was detected in the normal cell lines HEK293 and HK-2 by RT-PCR; *GAPDH* served as the control; and (**B**) *ADAMTS18* gene hypermethylation was detected in the ccRCC-derived cell lines A498, Caki-2, Ketr-3 and Osr by MSP; It was unmethylated in normal cell lines. M, methylated; U, unmethylated.

To assess whether the *ADAMTS18* gene was down-regulated in ccRCC tumor tissues, we initially determined its expression in ccRCC-derived cell lines using RT-PCR. We found that *ADAMTS18* expression was completely silenced in all of the ccRCC-derived cell lines but was present in 2 normal renal cell lines (HEK293 and HK-2), as shown in Figure 1A. These results indicate that *ADAMTS18* is frequently downregulated in ccRCC-derived cell lines.

### 2.2. Hypermethylation of ADAMTS18 in ccRCC-Derived Cell Lines

We then examined the methylation status of *ADAMTS18* in ccRCC-derived cell lines using MSP. As expected, *ADAMTS18* was hypermethylated in four of the five ccRCC-derived cell lines (80.0%), including A498, Caki-2, Ketr-3 and Osr, which was consistent with the silenced expression described above. In contrast, no methylation was detected in the HEK293 or HK-2 cell line (Figure 1B). These results are consistent with the previously described RT-PCR studies. Unmethylated bands were detected in all cell lines, which could be explained by the fact that only a subset of these CpG sites were methylated. Another possible reason could be that the characteristics of the cell lines were altered during the preparation process such that the population became heterogeneous, with some cells being hypermethylated and others showing normal methylation.

### 2.3. Activation of ADAMTS18 Gene Expression after Demethylation via Drug Treatment in ccRCC-Derived Cell Lines

**Figure 2:**
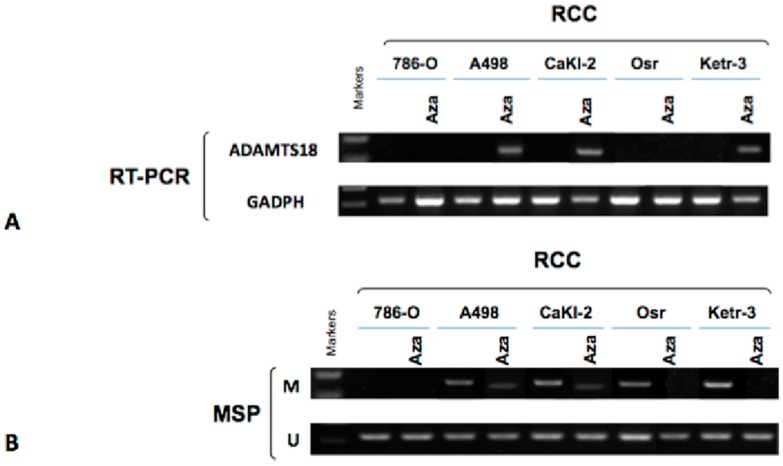
Analysis of the *ADAMTS18* gene in ccRCC-derived cell lines after drug treatment. (**A**) *ADAMTS18* gene expression was restored in the ccRCC-derived cell lines by 5-AzaC treatment, as observed by RT-PCR; *GAPDH* served as the control; and (**B**) The *ADAMTS18* gene was partially or completely demethylated in some of the ccRCC-derived cell lines by MSP. M, methylated; U, unmethylated.

To further determine whether hypermethylation directly mediated *ADAMTS18* gene silencing, we compared *ADAMTS18* gene expression levels in ccRCC-derived cell lines before and after three days of treatment with 5-AzaC using both RT-PCR and MSP. After the treatment, the* ADAMTS18* gene was expressed in three of the five (60%) ccRCC-derived cell lines; however, it was not expressed in the 786-O or Osr cell lines (Figure 2A). Meanwhile, MSP also showed that the *ADAMTS18* gene was partially or completely demethylated following pharmacological demethylation (Figure 2B). These changes further confirmed that *ADAMTS18* gene silencing is mediated by hypermethylation in ccRCC-derived cell lines.

### 2.4. Hypermethylation of the ADAMTS18 Gene in ccRCC Primary Tumors and Adjacent Normal Tissues

**Figure 3:**
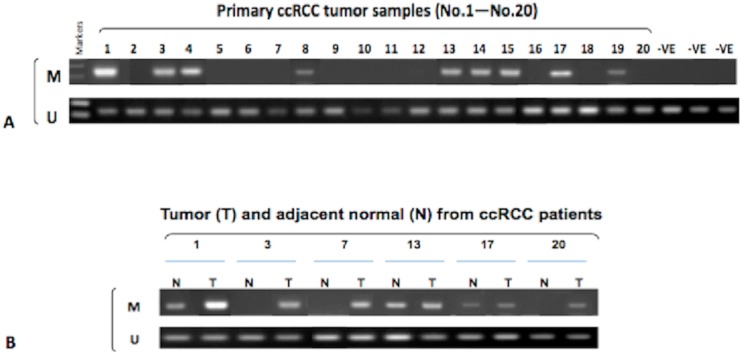
Representative MSP results. (**A**) *ADAMTS18* gene hypermethylation was detected in 9 of 20 (45.0%) primary tumors from the initial 20 cases (No. 1–No. 20); 3 non-cancerous normal tissues were used as negative controls; and (**B**) Paired ccRCC (T) and matched normal tissues (N) were compared, revealing 3 cases of *ADAMTS18* gene hypermethylation that appeared simultaneously in T and N. M, methylated; U, unmethylated.

We then used this validated system to analyze the methylation status of *ADAMTS18* in a series of 101 ccRCC samples and 20 adjacent normal tissues. Hypermethylation was detected in 44 of 101 (43.6%) primary tumors and 3 of 20 (15.0%) adjacent normal tissues; representative results are shown in Figure 3. However, a significant difference between the groups was observed (*p* = 0.02 < 0.05). Similar to the results obtained with ccRCC-derived cell lines, unmethylated bands were also detected in all ccRCC and nonmalignant samples, which could be explained by heterogeneity of the tumors and the infiltration of “normal” cells. Additionally, the matched tumor samples for the 3 adjacent normal tissue samples with *ADAMTS18* gene hypermethylation were also hypermethylated. This phenomenon highlights the importance of tumor-specific *ADAMTS18* gene hypermethylation in the tumorigenesis of ccRCC.

### 2.5. Bisulfite Genomic Sequencing (BGS) Analysis

**Figure 4:**
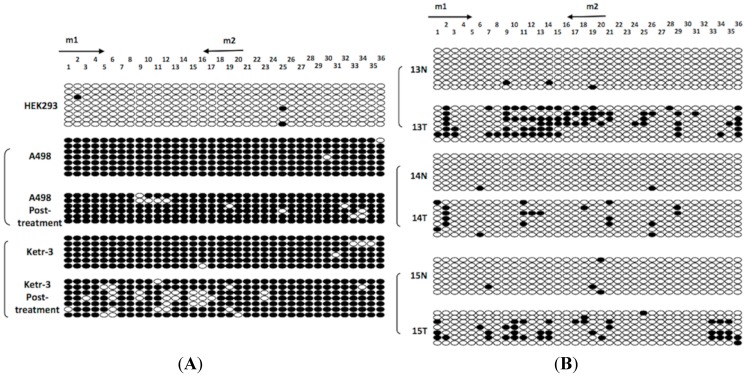
BGS high-resolution mapping of the methylation status of each CpG site (ovals) in the *ADAMTS18* gene promoter region. (**A**) BGS results for the HEK293, A498 and Ketr-3 cell lines; and (**B**) BGS results for the ccRCC primary tumors (T) and adjacent normal tissues (N). The rows represent the individual alleles of the *ADAMTS18* gene promoter that were analyzed. The arrows indicate MSP primer sites. The black circles indicate methylated sites, while the white circles indicate unmethylated sites.

Bisulfite genomic sequencing (BGS) was performed to confirm the methylation status of the *ADAMTS18* gene in the HEK293 and A498/Ketr-3 cell lines (before and after demethylation treatment) and in the three hypermethylated tumors along with their adjacent normal tissues. A typical CpG island in the *ADAMTS18* gene (an 1150-bp region containing 36 CpG sites) has been amplified and identified by Jin *et al.* [14]. The BGS results were consistent with the MSP results, revealing a high density of hypermethylated CpG sites in the A498/Ketr-3 cell lines (before demethylation treatment) and in the hypermethylated tumor samples (Figure 4A,B). However, not every CpG site in the *ADAMTS18* gene promoter region was hypermethylated, which indicated that methylation of only a subset of CpG sites could yield a positive MSP result, although unmethylated regions might be observed simultaneously.

### 2.6. Real-Time PCR Analysis

**Figure 5:**
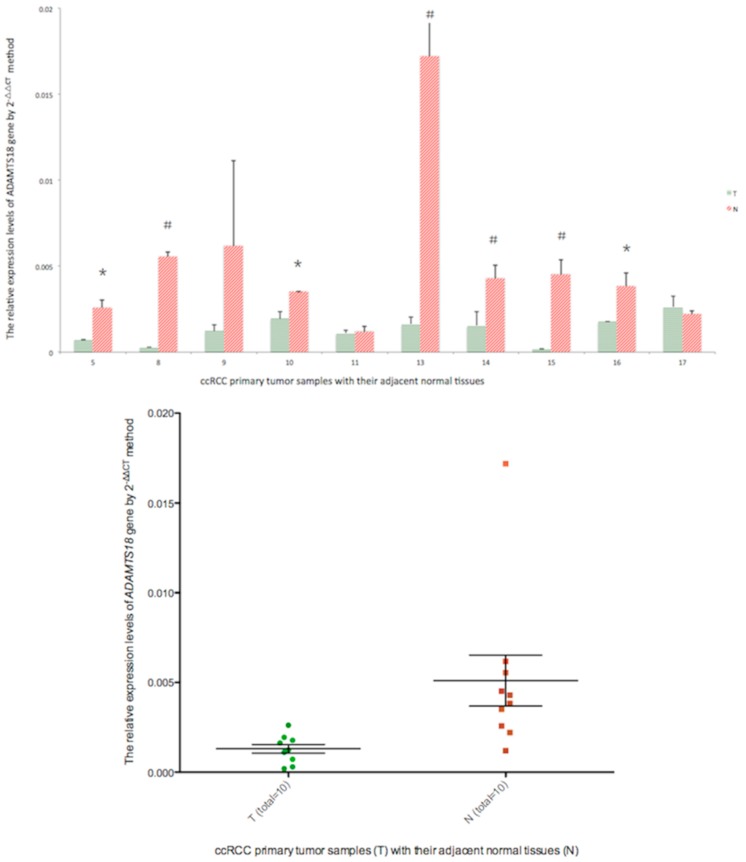
Real-time PCR results illustrating relative mRNA expression levels of *ADAMTS18* in 10 pairs of ccRCC primary tumor samples (green dots) compared with their adjacent normal tissues (red dots). * *p* < 0.05 and ^#^ *p* < 0.01.

To confirm that methylation of the *ADAMTS18* gene was indeed correlated with the down-regulation of its expression, we next performed real-time PCR to detect *ADAMTS18* mRNA expression levels in 10 primary tumors paired with their adjacent normal tissues (5T/N, 8T/N, 9T/N, 10T/N, 11T/N, 12T/N, 13T/N, 14T/N, 16T/N and 17T/N), as demonstrated in Figure 5. Numbers 6, 7 and 15 were excluded due to insufficient quantities of material for real-time PCR analysis following MSP analysis. As expected, the expression levels of the *ADAMTS18* gene were significantly lower in 7 out of 10 (70%) ccRCC primary tumors (5T, 8T, 10T, 13T, 14T, 15T and 16T) compared with the adjacent normal tissues, which was consistent with our MSP results. Because both 17T and 17N were hypermethylated, there were no significant differences in the *ADAMTS18* expression levels between 17T and 17N. MSP-negative results were obtained for some of the tumors that showed significantly different *ADAMTS18* expression compared with their paired normal tissues based on real-time PCR (e.g., 5T/N, 10T/N and 16T/N). One possible explanation for this result is that the MSP-targeted region may not have been fully methylated at all CpG sites. Most importantly, the mRNA expression levels of *ADAMTS18* in 10 ccRCC tumor samples were significantly lower than those in their adjacent normal tissues (*p* = 0.014, Figure 5).

### 2.7. Analysis of Clinicopathological Features

Table 1 lists the clinicopathological features of the patients. The methylation status of *ADAMTS18* was not significantly associated with gender, age, tumor location or tumor diameter. Additionally, there was no association between AJCC pathological stage and nuclear grade in patients with different *ADAMTS18* methylation patterns. In summary, no correlation was found between *ADAMTS18* methylation status and any clinicopathological feature. The percentage of hypermethylated ccRCC tumors was not increased significantly in ccRCC tumors at more advanced stages or grades.

We were able to follow up with 91 patients (response rate of 90.1%) who were involved in this study, and the follow-up times ranged from 8 to 16 months. In total, 10 patients died during the follow-up period (5 patients in the methylated group and 5 in the unmethylated group). The mean DFS for the *ADAMTS18*-methylated patients was not significantly shorter than that for the *ADAMTS18*-unmethylated patients (12.11 *versus* 11.49 months, *p* = 0.7930).

**Table 1:** Clinicopathological features of ccRCC patients and *ADAMTS18* gene methylation status.

Clinicopathological Features		No. Methylated (%)	No. Unmethylated (%)	*p* Value
Overall		44 (43.6%)	57 (56.4%)	–
Age (years)		60.6 ± 9.6	56.9 ± 12.1	0.092
Gender	M	35 (49.3%)	36 (50.7%)	0.074
	F	9 (30.0%)	21 (70.0%)	
Side	Left	27 (45.8%)	32 (54.2%)	0.597
	Right	17 (40.5%)	25 (59.5%)	
Tumor diameters (cm)		5.9 ± 1.6	5.2 ± 2.2	0.085
AJCC pathological stage	I	27 (39.1%)	42 (60.9%)	0.400
	II	3 (60.0%)	2 (40.0%)	
	III	14 (51.9%)	13 (48.1%)	
Nuclear grade	G1	11 (41.9%)	17 (58.1%)	0.502
	G2	30 (45.9%)	33 (54.1%)	
	G3	3 (33.3%)	7 (66.7%)	

## 3. Discussion

ccRCC is a type of urological malignant tumor for which there are few effective treatments. A full understanding of the molecular mechanisms and pathology of ccRCC may provide a basis for developing novel approaches for early diagnosis and more effective therapies. Cancer-specific epigenetic alterations resulting in TSG inactivation are frequent events in ccRCC and may be potential markers for early detection; however, few genes with reduced levels of hypermethylation have been reported in ccRCC. Thus, low levels of methylation may not be biologically relevant, and the identification of genes with higher levels of methylation in ccRCC is critical. In this study, we validated the methylation status of a critical 16q23.1 TSG, the *ADAMTS18* gene, in ccRCC-derived cell lines and primary tumors, and we evaluated the relationship between the methylation status of *ADAMTS18* and the clinicopathological features of patients with ccRCC.

The *ADAMTS18* family contains multiple domains, including a metalloproteinase catalytic domain with a reprolysin-type zinc-binding motif, a disintegrin-like domain, a central thrombospondin type 1 repeat domain, and five *C*-terminal thrombospondin type 1 repeat domains [13,15]. The *ADAMTS18* gene, which is a member of the *ADAMTS18* family, is located on chromosome 16q23. The loss of this locus has been demonstrated to occur frequently in multiple tumor types, indicating that it contains critical TSGs, such as the well-studied *WWOX*. In fact, detailed mechanisms underlying the epigenetic alterations of most *ADAMTS18* family proteins are still unknown, and only the epigenetic alterations of the *ADAMTS18* gene have been reported in multiple human tumors, including colorectal, brain, ovarian, breast, lung, head and neck, hepatocellular, oesophageal, gastric and nasopharyngeal carcinomas [14].

This is the first study to estimate the prevalence of *ADAMTS18* gene methylation in a large set of ccRCC tumor samples. Using RT-PCR and MSP, we discovered that the *ADAMTS18* gene is hypermethylated in both ccRCC-derived cell lines and primary tumors, indicating that this gene, which is a novel functional TSG, is frequently epigenetically inactivated in ccRCC. MSP is a sensitive and specific method for detecting DNA methylation in tumor samples, and allowing the rapid examination of multiple samples [16], which revealed that the *ADAMTS18* gene promoter was hypermethylated in 80% of the ccRCC-derived cell lines (with the exception of the 786-O cell line) and 43.6% of the ccRCC primary tumors. The higher methylation levels of the ccRCC-derived cell lines compared to the primary tumors indicate that some ccRCC-derived cell lines may have acquired *ADAMTS18* gene methylation during establishment or maintenance of the line. Some researchers have argued that the *ADAMTS18* methylation percentage in primary ccRCC primary tumors (43.6%) is not likely to be high enough to warrant the use of this gene as an effective marker, and the hypomethylation data were not convincing. However, the hypermethylation levels of most TSGs are relatively lower in ccRCCs (only approximately 10% to 30%) than in other solid tumors (approximately 60% to 80%). For some unknown reason, the methylation level in ccRCC is always too low, as a methylation level of only 30% can be suggestive of a “frequently” methylated ccRCC tumor-specific promoter region [2]. Therefore, due to the above characteristics of epigenetic alterations in ccRCC methylation, unmethylated regions may always be present in ccRCC primary tumors and ccRCC-derived lines, even if the cell lines are homogeneous. Of course, non-methylation-specific primers might also not be specific enough in ccRCC-derived cell lines or ccRCC primary tumors, although they have been shown to be quite specific in oesophageal, nasopharyngeal and several other carcinomas [14]. There are unmethylated alleles in all of the cell lines and primary tumors, which suggests that other transcriptional regulatory mechanisms, such as histone modification or transcriptional repression, may also contribute to this process. Thus, the MSP reaction may not be specific enough for unmethylated DNA. Hence, it is necessary to combine high-throughput methylation profiling assays with confirmatory assays and investigations to confirm transcriptional silencing in future analysis [17].

As shown in Figure 4, the very small decrease in the level of CpG methylation induced by treatment of the cell lines with 5-AzaC appeared to cause re-expression, whereas in the tumor samples, the silenced alleles appeared to be methylated at a few CpG sites. We postulate that some “key sites” may exist in the promoter region, and the demethylating agent does not exert a complete effect; rather, it serves as an important tool that can be used to restore partial protein function by targeting CpG sites. Indeed, we postulate that only these “key sites” can directly mediate the methylation of the promoter region. Therefore, the demethylating agent is only likely to play a crucial role in protein re-expression when the “key sites” of the promoter region are demethylated. Otherwise, protein re-expression will be impossible to realise, even if most of the CpG sites are unmethylated. Additionally, there may not be a single CpG site that can explain this extraordinary phenomenon by itself. In future experiments, we will further investigate whether these “key sites” actually exist in the promoter region using a large-scale analysis involving the application of demethylating agents to hypermethylated tissue samples.

Peters *et al.* has assumed that a variety of epigenetic alterations contribute at different stages of carcinogenesis to the development of RCC [18]. However, surprisingly, no association was observed between the methylation status of the *ADAMTS18* gene and the clinicopathological features of the patients with ccRCC, which is supported by the hypothesis that *ADAMTS18* gene hypermethylation is a cause, rather than a consequence, of carcinogenesis. Thus, even in stage I ccRCC, the *ADAMTS18* gene is already hypermethylated. Similar findings have also been noted for *PCDH8* methylation, which also occurred in early-stage ccRCC tumors and some adjacent normal renal tissues, indicating that *PCDH8* methylation may be correlated with the initiation and progression of ccRCC [19]. We further demonstrated that hypermethylation of the *ADAMTS18* gene was not significantly associated with shorter disease-free survival suggesting that it is not an independent prognostic factor in patients with ccRCC. Therefore, the *ADAMTS18* gene may not be a suitable marker for the stage, grade or survival of ccRCC.

Surprisingly, *ADAMTS18* gene hypermethylation was also detected in some adjacent normal tissues. Hypermethylation of *ADAMTS18* was not detected in the non-tumor cell lines or normal renal parenchyma specimens, although our investigation revealed that 15.0% of the adjacent normal tissues were hypermethylated. More importantly, the paired tumor samples of these 3 adjacent normal tissues were also hypermethylated, indicating that *ADAMTS18* gene analysis may be useful in diagnosing ccRCC. Promoter methylation in adjacent normal tissues can be caused by various factors related to diet or aging [20], which may result in low methylation frequencies in normal tissues. Remco *et al.* [21], have reported that the methylation of normal tissues may be the result of environmental insults and aging, indicating that physiological and epigenetic regulation is ongoing in certain cell types in normal tissues. Wistuba [22], has reported that methylation might occur as part of a premalignant field effect. There is no statistical evidence to indicate that hypermethylation is more likely to occur in advanced-stage or high-grade ccRCC, either in ccRCC tumor samples or adjacent normal tissues.

Unfortunately, no detailed information is available on the functional effects of *ADAMTS18* gene hypermethylation. Additionally, both the limited sample size and short-term follow-up information have made it difficult to draw an accurate conclusion concerning the significance of *ADAMTS18* gene hypermethylation, and these parameters will be improved. Only a large-scale analysis with a long-term follow-up period can accurately determine whether *ADAMTS18* gene hypermethylation can be applied as a new biomarker for ccRCC.

## 4. Materials and Methods

### 4.1. Patients and Tissue Samples

All human primary ccRCCs (101 cases) and adjacent normal tissues (20 cases) were obtained from the Urology Department of Peking University First Hospital in Beijing from September 2012 to May 2013. Specimens were collected by radical or partial nephrectomy, and ccRCC diagnoses were confirmed by pathological findings. The adjacent normal tissues were resected at least 2 cm from the tumors, and histologically tumor-free margins were subsequently verified by two experienced pathologists. Additionally, three similarly aged normal renal parenchyma specimens were collected during non-cancer-related kidney surgery that was performed during the same time period for use as normal controls. These specimens were also examined pathologically to exclude the possibility of incidental tumors. The inclusion criterion for control subjects was no prior history of any malignant tumors. All of the resected tissues were snap-frozen in liquid nitrogen and stored at −80 °C. Patients with localised lymph node or distant metastases detected by preoperative computed tomography scan were excluded from this study.

The tumor set comprised 71 males and 30 females ranging in age from 28 to 78 years with a median age of 58.5 years at diagnosis. In 58 patients, the primary tumors were located on the left side, and in 43 patients, the primary tumors were located on the right side. The average preoperative diameter of the primary tumors was 5.5 cm (range, 2.1–12.5 cm). The classifications of the tumors were classified based on the staging system of the 2009 American Joint Committee on Cancer (AJCC). Additionally, the nuclear grades of the tumors were determined during postoperative pathological analysis.

### 4.2. Cell Line Preparation and Treatment of ccRCC-Derived Cell Lines with a Demethylating Drug

Five ccRCC-derived cell lines (A498, Caki-2, Ketr-3, Osr, and 786-O) obtained from the American Type Culture Collection (ATCC, Manassas, VA, USA) were prepared to validate the methylation status of *ADAMTS18*. The HEK293 human normal embryonic kidney cell line and HK-2 human kidney proximal tubular epithelial cell line were both routinely cultured and served as “normal” controls. All of these cell lines were maintained in dulbecco’s modified eagle medium supplemented with 2 mM glutamine and 10% fetal bovine serum at 37 °C with 5% CO_2_. The demethylating agent 5-AzaC (Sigma^®^, Hong Kong, China) was freshly prepared in ddH_2_O and filter sterilised. Then, the ccRCC-derived cell lines were demethylated by daily treatments with fresh medium containing 10 µM 5-AzaC for 3 days (doubling time: 36 h). The medium was changed every 24 h. After the treatment, the cells were pelleted and washed with PBS, and DNA/RNA was extracted.

### 4.3. Semi-Quantitative Reverse Transcription PCR (RT-PCR)

*ADAMTS18* gene expression in the ccRCC-derived cell lines before and after treatment with 5-AzaC was determined by RT-PCR using an RNA PCR kit (Applied Biosystems^®^, Shanghai, China) and Go Taq^®^ (Shanghai, China). The 12.5-µL RT-PCR mixture contained 2.5 µL of RT product, 0.75 µL of 5' primer (10 µM), 0.75 µL of 3' primer (10 µM), 2 µL of 5× Flexi buffer, 0.5 µL of MgCl_2_ (25 mM), 0.0625 µL of Promega Go Taq^®^ (5 U/µL) and 5.9375 µL of ddH_2_O. *GADPH* was used as an internal control. Table 2 lists the primer sequences and the cycling parameters for RT-PCR analysis.

**Table 2:** The sequences and conditions of PCR primers.

Gene	PCR	Primer Sequences (5'→3')	Annealing Temperature (°C)	Cycles
*ADAMTS18*	RT-PCR (real-time PCR)	F: TAGCCAGTGACAGCAGCAG	55	37
		R: CTAAGTGCAGTTCCTGTCCA	55	37
	MSP	m1: TTGTAGTTCGGTAGGTTCGC	60	40
		m2: ACTCCAAATAAAAACCGCCG		
		u1: AAATTGTAGTTTGGTAGGTTTGT	58	38
		u2: CAACTCCAAATAAAAACCACCA		
	BGS	BGS1: GTTTTAGTTTYGGTTTAGGGAGTT	60	40
		BGS2: AACRCACTCCATAATCAAATAC		

### 4.4. DNA Extraction from ccRCC Tissue Samples

Genomic DNA was extracted from 101 ccRCC tissues and 20 adjacent normal tissues using the TIANamp Genomic DNA Kit (TIANGEN^®^, Shanghai, China) according to the manufacturer’s protocol. The quality of the isolated DNA was subsequently assessed by electrophoresis.

### 4.5. Bisulfite Modification of DNA and Methylation-Specific PCR (MSP)

Bisulfite modification of DNA and MSP were carried out as described previously [11]. Then, to identify epigenetic alterations in *ADAMTS18* in ccRCC primary tumors, bisulfite-modified DNA (50 µg) was used in each MSP system (Qian Tao, Hong Kong, China) with primers specific for methylated DNA and primers specific for unmethylated DNA. All of the primers were provided by Sangon Biotech^®^ Co., Ltd. (Shanghai, China) and were previously shown to be unable to amplify non-bisulfite-modified DNA. The MSP primer sequences, annealing temperatures and cycling details are described in Table 2. Each 12.5-µL MSP mixture contained 0.5 µL of bisulphate-treated genomic DNA, 1 µL of dNTPs (2.5 mM), 0.75 µL of 5' primer (10 µM), 0.75 µL of 3' primer (10 µM), 2 µL of 5× Flexi buffer, 1 µL of MgCl_2_ (25 mM), 0.09375 µL of AmpliTaq Gold^®^ (5 U/µL) and 6.40625 µL of ddH_2_O. The MSP products were run on a 2% agarose gel together with a molecular weight marker, and the gel was subsequently stained with ethidium bromide.

### 4.6. Bisulfite Genomic Sequencing (BGS) of ADAMTS18 in Cell Lines and Specimens

BGS was performed to determine the methylation status of *ADAMTS18* in several cell lines and specimens following methods described in a previous study by Zhang *et al.* [11]. The BGS primers used are listed in Table 2. Amplified BGS products were TA-cloned, and six colonies were chosen and sequenced. At least five individual colonies were collected to ensure a representative sample [10,11,14,15,23,24].

### 4.7. Quantitative Real-Time PCR

Total RNA from 10 paired tumor and adjacent normal tissue samples was isolated using Trizol reagent (Invitrogen, Carlsbad, CA, USA), and 2 µg of total RNA was reverse-transcribed using the Reverse Transcription System (Qian Tao, Hong Kong, China). Real-time PCR was performed using the ABI Prism 7500™ instrument (Applied Biosystems) with SYBR Green PCR Mix. *GAPDH* was used as the internal control. Each reaction was performed with specific primer sets (Table 2). The relative expression levels of the *ADAMTS18* gene were calculated according to the 2^−∆**∆***C*t^ method.

### 4.8. Statistical Analysis

Clinicopathological data of the 101 ccRCC patients were obtained from our institutional database. The *ADAMTS18* methylation status and the clinicopathological features of the ccRCC patients were analyzed. Disease-free survival (DFS) was measured from the day of operation until the day of recurrence or the most recent follow-up visit. Continuous data are shown as the mean ± standard deviation (SD). All of the statistical analyses were performed using Student’s *t* test, Fisher’s exact test or the chi-square test with SPSS 17.0 software (StatSoft Inc., Tulsa, OK, USA). A *p* value <0.05 was considered statistically significant.

## 5. Conclusions

We report for the first time that the *ADAMTS18* gene is often downregulated by hypermethylation in ccRCC-derived cell lines and primary tumors, indicating that it plays a critical role as a TSG in ccRCC. Thus, we conclude that *ADAMTS18* gene hypermethylation may be involved in the tumorigenesis of ccRCC. While further investigation is required, this hypermethylation status may be considered a potential novel biomarker for this disease when detected in the serum and urine samples of ccRCC patients.
